# Effect of Metformin Intervention during Pregnancy on the Gestational Diabetes Mellitus in Women with Polycystic Ovary Syndrome: A Systematic Review and Meta-Analysis

**DOI:** 10.1155/2014/381231

**Published:** 2014-05-21

**Authors:** Zhihong Zhuo, Aiming Wang, Huimin Yu

**Affiliations:** ^1^Southern Medical University, Guangzhou 510000, China; ^2^Ningbo No. 2 Hospital, Ningbo 315010, China; ^3^Navy General Hospital of Chinese PLA, Beijing 100000, China

## Abstract

Metformin is an effective insulin sensitizer treating type 2 diabetes mellitus. However, the functional consequences of metformin administration throughout pregnancy on gestational diabetes mellitus (GDM) with polycystic ovary syndrome (PCOS) have not been assessed. We therefore performed a meta-analysis and system review to determine the effect of metformin on GDM in PCOS. A meta-analysis was performed on the published studies before December, 2013. Meta-analysis examined whether metformin could reduce GDM occurrence in PCOS with a fixed effect model. The odds ratio (OR) with 95% confidence interval (95% CI) was calculated to estimate the strength of association. A total of 13 studies including 5 RCTs and 8 non-RCTs were enrolled. Ultimately, effectiveness analysis demonstrated that, in total, there was no significant availability of metformin on GDM in PCOS in contrast to placebo (OR = 1.07, 95% CI 0.60–1.92) in RCTs and significant availability of metformin on GDM (OR = 0.19, 95% CI 0.13–0.27) was indicated in non-RCTs. In summary, according to the results of our meta-analysis, strictly, metformin did not significantly effect on GDM with PCOS, though more multicenters RCTs still need to be investigated.

## 1. Introduction

Polycystic ovarian syndrome (PCOS), which is one of the common endocrine disorders, is one of the main causes of ovulatory infertility, affecting 5–10% of women of reproductive age [1, 2]. PCOS is characterized by the presence of typical ultrasound features of polycystic ovaries, oligomenorrhea, and clinical and/or biochemical hyperandrogenism and, commonly, by insulin resistance, hyperinsulinemia, morbid obesity, and infertility [3–9]. Insulin resistance appears in both obese and nonobese women with PCOS [10]. Among these women, insulin promotes intraovarian steroidogenesis by interacting with luteinizing hormone (LH) leading to inappropriate advancement of granulose cell differentiation and arrest of follicle growth. The outcomes with hyperinsulinemia may directly enhance ovarian secretion and abnormal follicular development, which ultimately lead to ovarian dysfunction [11, 12]. Moreover, hyperinsulinemia has been suggested as pathogenic factors in pregnancy complications [6]. Insulin resistance or obesity with PCOS leads to a significant increase in gestational outcomes and difficulties during labor.

During recent years, metformin, which is an effective oral biguanide insulin sensitizer, has been widely used for treating type 2 diabetes mellitus (T2DM) as an antihyperglycemic agent [13], by improving tissue sensitivity to insulin while inhibiting hepatic glucose production, enhancing peripheral glucose uptake, and decreasing insulin levels [14, 15], and approved by the United States Food and Drug Administration (FDA) [16]. Therefore, metformin has become an ideal first line therapy for individuals with T2DM. When treated in women with PCOS, especially these patients with hyperinsulinemia, metformin corrects hyperinsulinemia and also reduces ovarian androgens, LH and sex hormone binding globulins. Metformin has been increasingly regarded to be effective and safe medicine for the metabolic and endocrine abnormalities in PCOS [17, 18]. Its use as a drug for ovulation induction in PCOS has been extensively investigated and has been found to increase the likelihood of ovulation and decrease miscarriage rates, particularly in patients who have clomiphene resistance before. As we all know, metformin is a category B drug for use in pregnancy (absence of teratogenic effects based on animal data) and its characteristics of effectiveness and safety and, as a result, its use in pregnancy have become increasingly popular worldwide, although there are no guidelines for its continuous use in pregnancy and there is debating on potential adverse effects on both the mother and the fetus because of its crossing the placenta [19, 20].

Moreover, metformin has been considered as a potentially effective agent during pregnancy to treat gestational diabetes mellitus [21]. On the other hand, several randomized controlled trials (RCTs) evaluating the reproductive effect of metformin administration and reporting the occurrence of GDM are not available in literatures. Experimental and clinical studies seem to suggest that metformin does not have any effect on the incidence of GDM in women with PCOS. Furthermore, unclear and nonpowered data support the use of metformin for the prevention of GDM, preeclampsia, and other gestational complications in PCOS patients [22].

Based on these considerations, we conducted a systematic review and meta-analysis of trials for a more objective appraisal of evidence regarding the effect of pregestational metformin administration on gestational complications, especially the gestational diabetes in women with PCOS.

## 2. Materials and Methods

A systematic review was conducted with all pertinent studies that were found in the electronic databases MEDLINE, EMBASE, and the Cochrane Central Register of Controlled Trials (CENTRAL) that examined metformin and pregnancy outcome on the women with PCOS from 1966 to December, 2013. The search strategy included the terms* pregnancy, pregnant complication, GDM, gestational diabetes mellitus, diabetes, metformin, biguanide, insulin-sensitizing drugs, insulin-sensitizers, polycystic ovary syndrome*, and* PCOS*. The search was concluded by (1) the perusal of the reference sections of all relevant studies in all languages and (2) a manual search of the key journals and abstracts from the major annual meetings in the fields of endocrinology and obstetrics and gynecology. Articles were excluded from the analysis if they did not have either adequate disease-matched control groups or data on the outcome of the pregnancy with respect to major gestational diabetes and exposure to metformin throughout pregnancy or in at least the first trimester. The control groups consisted of women with PCOS who were not treated with metformin or placebo.

The inclusion and exclusion criteria were as follows.

Inclusion criteria:

1. PCOS was exposed to metformin throughout the pregnant period, or at least during first trimester;
2. studies could be a prospective, retrospective, or a case-control study; however, there had to be a control group;
3. studies were written in any language.

Exclusion criteria:

1. if type of articles was review or letter to the editor;
2. animal studies;
3. studies with no control group or with an inappropriate control group;
4. women with exposures to other known pathogenic factors or other maternal disorders that might affect the outcome.

Titles and, especially, abstracts were screened, and the potential relevant researches were identified. Two independent reviewers (ZHUO and YU), who were not blinded to the names of investigators or sources of publications, identified and selected the articles that met the inclusion and exclusion criteria below. The reviewers worked independently and in duplicate. Disagreements between two of them were resolved by consensus or arbitration between each other. Meta-analysis was conducted using Review Manager 5.2 software (Review Manager (RevMan) (computer program) (Version 5.2 for Windows), Oxford, England: The Cochrane Collaboration, 2012).

We included trials evaluating the reproductive effects of metformin administration on the gestational complications especially GDM in the women with PCOS in the current meta-analysis. We considered all studies in which the diagnosis of PCOS was based on the normal criteria such as Rotterdam ESHRE/ASRM consensus criteria, NIH criteria, or AES criteria eligible.

The quality of the studies included was evaluated by the Cochrane guidelines [23]. Specifically, allocation concealment, blinding, intention-to-treat (ITT) analysis, and followup were assessed for each trial. Allocation concealment was graded as adequate (A), unclear (B), or inadequate (C) according to the criteria provided by the Cochrane Group [24] particularly. Blinding was reported as yes, no, or not reported for patients, outcome assessors, investigators, or data analysts. The use of ITT analysis was recorded and indicated as yes or no.

The *Q*-test of Cochran RevMan was used to measure the heterogeneity between articles and was calculated as the weighted sum of squared differences between individual study effects. A Cochran *Q*-test *P* ⩽ 0.05 represents a statistical homogeneity. For data with statistical homogeneity, the Mantel-Haenszel method was used to calculate the weighted summary RR under the fixed effects model. On the other hand, the random-effect model of meta-analysis was used in the presence of unexplained statistical heterogeneity. *P* ⩽ 0.05 of 95% CI not containing 1.0 RR was considered statistically significant.

## 3. Results

### 3.1. Flowchart of Study Selection

**Figure 1:**
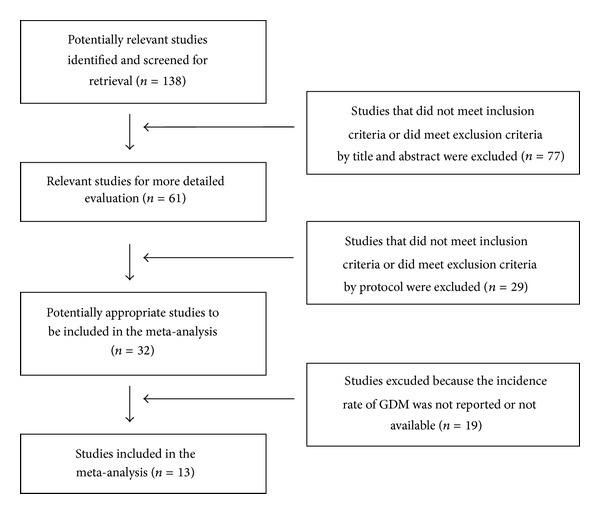
Flowchart of the study selection.

The flowchart of the study selection according to the inclusion and exclusion criteria above is shown in Figure 1. A total of 13 trials potentially resulted for inclusion in the meta-analysis [22, 25–36]. In particular, we excluded the trials as follows: the data on the occurrence of GDM were not available from papers and could not be obtained from the investigators by e-mail contact; it was not possible to contact the corresponding investigators and the occurrence rate of gestational diabetes has not been evaluated.

### 3.2. Characteristics of Studies

Table 1 summarizes the quality of the trials included in the analysis. The main characteristics of the populations, the interventions received, and the outcomes obtained in the trials are summarized in Tables 2 and 4, respectively. A total of five RCTs were also included in the final analysis as Table 3. However, three RCTs including Fougner et al. [30], Vanky et al. [32], and Salvesen et al. [35] are duplicate, and Vanky et al. [22] reported an epianalysis of two randomized controlled trials as Vanky et al. [32] and Salvesen et al. [35]. Finally, we reviewed two RCTs [32, 35] in our meta-analysis. No research included in the system review and meta-analysis had occurrence of gestational diabetes as a primary end point, and none was powered to detect differences in GDM incidence. In addition, other potential gestational complications, such as miscarriage, preeclampsia, and preterm delivery, including the infants' characteristics, were investigated in these studies. The populations studied in the different trials were heterogeneous for several demographic and biochemical/metabolic characteristics.

### 3.3. Meta-Analysis

**Figure 2:**
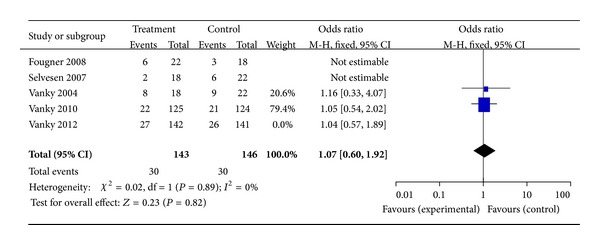
The meta-analysis of the odds ratio for gestational diabetes in RCTs.

**Figure 3:**
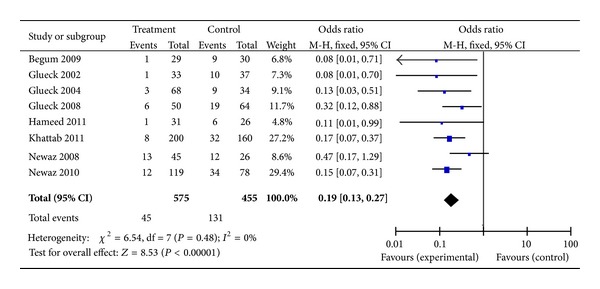
The meta-analysis of the odds ratio for gestational diabetes in the studies with disease-matched controls or patients' selves as control.

**Figure 4:**
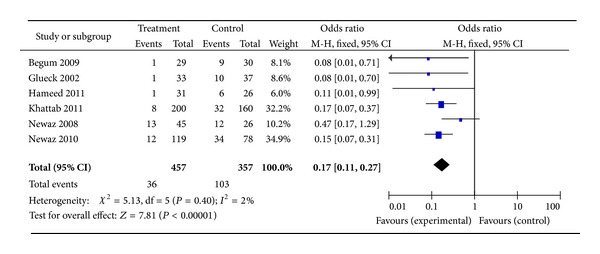
The meta-analysis of the odds ratio for gestational diabetes in the studies with PCOS as control.

**Figure 5:**
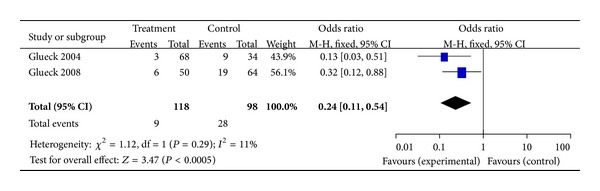
The meta-analysis of the odds ratio for gestational diabetes in the studies with patients' selves as control.

13 studies met the inclusion criteria, including 5 random control trial studies in the calculation of overall GDM occurrences. Exposure to metformin throughout the pregnancy was not associated with a decreased rate of GDM occurrence. The OR for GDM in RCTs with 289 subjects was 1.07 (95% CI 0.60–1.92, *P* = 0.89, and *I* ^2^ = 0%) (Figure 2) (Fougner et al. [30] and Selvesen et al.'s studies [35] were eliminated but Vanky et al.'s study [32] was enrolled because of the same object of three RCTs; meanwhile, Vanky et al.'s study [22] was eliminated because of the fact that it is an epianalysis of Vanky et al. [26] and Vanky et al. [32]); inversely, the OR including the studies with disease-matched controls or patient self as control was 0.19 (95% confidence interval (CI), 0.13–0.27,  *P* = 0.48,  *I* ^2^ = 0%) (Figure 3). Subanalysis was conducted to separate studies into those with PCOS and those with patients' selves with PCOS subjects as controls (Figures 4 and 5, resp.). With 457 subjects in the treatment group, the OR for the nondiabetes pregnancy subjects group was 0.17 (95% CI, 0.11–0.27, *P* = 0.40,  *I* ^2^ = 2%). With 118 subjects in the treatment group for PCOS with self as control, the OR was 0.24 (95% CI, 0.11–0.54, *P* = 0.29, and *I* ^2^ = 11%).

## 4. Discussion

In our mind, this is the first systematic review and meta-analysis of trials including RCTs performed to establish the potential effect of metformin invention during the gestational period on gestational diabetes occurrence risk in patients with polycystic ovary syndrome.

Gestational diabetes mellitus (GDM) represents an important medical and social problem in the general pregnancy population, and it becomes harmful to the health of mother and fetus as well as neonate. Boomsma et al. [37] investigated that women with PCOS demonstrated a significantly higher risk of developing GDM (OR = 2.94), pregnancy-induced hypertension (OR = 3.67), and preeclampsia (OR = 3.47), and their babies had a significantly higher risk of admission to a neonatal intensive care unit (OR = 2.31) and a higher perinatal mortality (OR = 3.07). Thiebaugeorges et al. [38] has included that the traditional therapy in pregnancy for GDM includes the use of diet and exercise and/or insulin injection. Meanwhile, several researchers have already demonstrated that the administration of metformin throughout pregnancy may decrease miscarriage and improve pregnancy outcomes including GDM, preeclampsia, and preterm delivery [34, 39–44].

Although a population-based cohort research, a system review, and meta-analysis recently identified that the patients with PCOS are at higher risk for pregnancy complications [45, 46], moreover, unless metformin was recommended for treatment in pregnancy complications especially DM because of its characteristics of safety, effectiveness, and nonteratogenicity, they could not reach an agreement on its use in prevention or treatment of GDM, hyperinsulinemia, and hyperandrogenemia throughout the pregnancy in women with PCOS. In our knowledge, during the past several decades, the retrospective and nonrandomized studies have confirmed beneficial effects of metformin on pregnancy miscarriage and pregnancy complications, in particular GDM in women with PCOS, whereas the potential effect of metformin to pregnancy complications in women with PCOS has been explored in five randomized, placebo-controlled trials so far [26, 32]. The studies suggested no statistical difference in the prevalence of gestational diabetes, preeclampsia, and preterm delivery between the metformin- and the placebo-treated groups. As a result, meta-analytical summaries of existing studies therefore are of importance to GDM in PCOS.

Although it yields a reassuring trend, this analysis exemplifies the need for more research on metformin to DM during pregnancy. It was our initial aim to identify all possible adverse pregnancy outcomes including miscarriage, minor anomalies, major malformations, intrauterine growth retardation, GDM, preterm delivery, live birth, caesarean section rate, or the characteristics of infants in particular the birth-height, birth-weight, APGAR in 1 or 5 or 10 minutes, and head circumference. However, we were limited to focusing mainly on the pregnancy complications on GDM because of the paucity of data and the paradoxical effect of metformin on GDM in PCOS.

Notwithstanding our data, metformin administered alone throughout the pregnancy, or at least during first trimester, or in combination with other infertility treatments for inducing ovulation in patients with PCOS seems to have significant effect on GDM prevention. In fact, our non-RCTs data demonstrated reduction of GDM occurrence risk in patients who received metformin in comparison with those who did not, for an overall relative risk for GDM of 0.19 (95% CI, 0.13–0.27). These findings were also confirmed by categorizing studies according to the objects control. Nevertheless, on the basis of RCT studies available now, metformin does not appear to be unavailable for use during pregnancy with respect to diabetes mellitus, for an overall relative risk for GDM of 1.07 (95% CI, 0.60–1.92). As with placebo use in pregnancy, it is challenging to establish the necessity of metformin use in pregnancy considering the background incidence of diabetes in the confounder of the underlying diseases with PCOS that have been linked to increased rates of diabetes.

However, it is not always true that RCTs and non-RCTs arrive at the same conclusions and, consequently, it is important to ask what should be done when they do not agree. First of all, the evidence of data derived from multiple randomized clinical trials or meta-analyses is stronger than data derived from a single randomized trial or unrandomized studies [47]. Thus, if a RCT study differs from several non-RCTs, then there is a pressing need to identify the studies much more deeply. We examine the possible reasons why two types of studies arrived at different conclusions and the potential threats to validity of observational outcomes and RCTs. Our analysis and review indicate that several methodological issues are relevant because they may affect the interpretation of some of the studies published on GDM. Most of the studies were retrospective or prospective of cohorts or case-control studies or observation researches and conducted at single center or multiple centers, and many studies included a small number of women, or else, the five RCTs, including the three studies of the same research object and one study epianalyzing the other two trials, took place in the same country as Norway and the study group of patients mainly recruited from the near clinic center imposed the restrictions on the outcomes, and thus the current situation limited the validity of the results. The second factors of different conclusion may be selection bias. Perhaps the most serious advantages of RCT are blindness and randomization because there may be large observed and unobserved differences in patients between the treatment and control groups [48]. These differences may explain why our meta-analysis concludes different results of RCTs and non-RCTs as RCTs were developed for the purpose of eliminating this bias.

During pregnancy, extra care must be taken to ensure the safety of the fetus or neonates. Our meta-analytical summary and review of inclusive studies therefore have indicated monitoring metformin safety. Although all included studies are very heterogeneous for protocols and doses of the drug administered and for characteristics of the studied populations, the detected observation of the results, such as lactic acidosis, birth defects, and maternal or neonatal hypoglycemia, is just a case-report and has no statistical analytical significance.

Our systematic review and meta-analysis of RCTs studies demonstrates no statistically significant benefit of metformin administration on the DM risk in PCOS patients who received the drug as monotherapy. However, we examine that non-RCTs have a characteristic of a lower GDM occurrence rate in the metformin group compared with the PCOS-matched control group. Thus, at the moment, there is no clinical evidence to suggest that metformin administration reduces the risk of diabetes in women with PCOS.

## 5. Conclusion

Metformin is an effective insulin sensitizer treating type 2 diabetes mellitus. However, the functional consequences of metformin administration throughout pregnancy on gestational diabetes mellitus (GDM) have not been assessed. Metformin has been increasingly regarded to be effective and safe medicine for the metabolic and endocrine abnormalities in PCOS. In our knowledge, this is the first systematic review and meta-analysis of trials including RCTs performed to establish the potential effect of metformin intervention during the gestational period on gestational diabetes occurrence risk in patients with polycystic ovary syndrome. We therefore performed a meta-analysis and system review to determine the effect of metformin on GDM in patients with PCOS. A meta-analysis was performed on the published studies before December, 2013. Meta-analysis examined whether metformin could reduce GDM occurrence in PCOS with a fixed effect model. The odds ratio (OR) with 95% confidence interval (95% CI) was calculated to estimate the strength of association. A total of 13 studies including five RCTs and eight non-RCTs were enrolled. Ultimately, effectiveness analysis demonstrated that, in total, there was no significant availability of metformin on GDM in PCOS in contrast to placebo (OR = 1.07, 95% CI 0.60–1.92) in RCTs and significant availability of metformin on GDM (OR = 0.19, 95% CI 0.13–0.27) was indicated in non-RCTs. Subanalysis was conducted to separate studies into those with PCOS and those with patients' selves with PCOS subjects as controls. With 457 subjects in the treatment group, the OR for the non-diabetes pregnancy subjects group was 0.17 (95% CI, 0.11–0.28). With 118 subjects in the treatment group for PCOS with self as control, the OR was 0.24 (95% CI, 0.11–0.54). In summary, according to the results of our meta-analysis, strictly, metformin did not significantly have effect on GDM with PCOS, though more multicenters RCTs still need to be investigated. Thus, at the moment, there is no clinical evidence to suggest that metformin administration reduces the risk of diabetes in women with PCOS.

## Figures and Tables

**Table 1:** Metformin studies included in the meta-analysis.

Number	Authors	Author study type	Data collection	Malformation described
22	Vanky et al., 2012 [22]	CO (RCT with placebo)	P	No description.
25	Khattab et al., 2011 [25]	CO	P	No miscarriages or neonatal loss occurred in either group. One baby girl born with major birth defect (tracheoesophageal fistula).
26	Vanky et al., 2010 [26]	CO (RCT with placebo)	P	Metformin group: one postpartum pulmonary embolism, one postpartum circulatory shock, one peripartum cardiomyopathy, and one sudden infant death. Placebo group: three spontaneous abortions, ileus in one patient with former gastric bypass operation, and one perinatal infant death caused by serious asphyxia.
27	Nawaz and Rizvi, 2010 [27]	CC	P	No description.
28	Begum et al., 2009 [28]	CO	P	No description.
29	Nawaz et al., 2008 [29]	CC	P	No stillbirths, perinatal deaths, or major birth defects. One baby in group A had polydactyl.
30	Fougner et al., 2008 [30]	CO (RCT with placebo)	P	No description.
31	Glueck et al., 2008 [31]	CO (self as control)	P	No maternal lactic acidosis and no maternal or neonatal hypoglycemia. Of the 180 live births to the 142 nondiabetic women with PCOS, there was one major birth defect (sacrococcygeal teratoma), as determined by pediatricians without knowledge of metformin dose or duration.
32	Vanky et al., 2004 [32]	CO (RCT with placebo)	P	No description.
33	Glueck et al., 2004 [33]	CO (self as control)	P	No maternal lactic acidosis or maternal hypoglycemia; no major birth defects; there have been no congenital defects or evidence of intrauterine growth. Seventy of the 84 fetuses have had a favourable outcome; no cases of neonatal hypoglycemia.
34	Glueck et al., 2002 [34]	CO	P and R	None developed lactic acidosis. Intermittent diarrhea or gastritis was common in the first 3 weeks of metformin therapy but resolved spontaneously and was not limiting factors. No major fetal malformations or fetal hypoglycemia occurred.
35	Salvesen et al., 2007 [35]	CO (RCT with placebo)	P	No description.
36	Hameed et al., 2011 [36]	CO	P	delivered spontaneously at 33 weeks gestation with neonatal asphyxia due to respiratory distress syndrome and died 2 days later.

Note: CO: cohort; CC: case-control; P: prospective; R: retrospective.

**Table 2:** Characteristics of trials in the meta-analysis.

Number	Population	Intervention	Comparison	Relevant outcomes
22	313 singleton pregnancies in women aged 18–45 years with PCOS (Rotterdam criteria).	Metformin (850 mg twice daily or 1000 mg twice daily), *n* = 153. Age 29.5 ± 4.4, BMI 29.8 ± 7	160 PCOS. Age 29.1 ± 4.3, BMI 28.6 ± 7.3.	Second-trimester miscarriage, preterm delivery, preeclampsia, and gestational diabetes.

25	360 nondiabetic PCOS patients (Egypt) (Rotterdam criteria) included were taking metformin for 3–6 months before they became pregnant.	200 pregnant women continued on metformin at a dose of 1000–2000 mg daily throughout pregnancy. Age 30.8 ± 2.2, BMI 30.1 ± 1.5.	160 women discontinued metformin use at the time of conception. Age 31.5 ± 2.4, BMI 29.6 ± 1.6.	Gestational diabetes, preeclampsia, and caesarean section rate.

26	274 pregnancies were randomly assigned to either metformin or placebo treatment. Criteria were (1) PCOS diagnosed according to the Rotterdam criteria, (2) age 18–45 yr, (3) gestational age between 5 and 12 wk, and (4) a singleton viable fetus shown on ultrasonography.	The metformin (2000 mg daily) treatment in pregnant PCOS women (*n* = 135). Age 29.6 ± 4.4, BMI 29.5 ± 7.0.	Placebo in pregnant PCOS women (*n* = 138). Age 29.2 ± 4.4, BMI 28.5 ± 7.2.	Preeclampsia, preterm delivery, GDM weight, blood pressure, heart rate, and mode and length of delivery.

27	197 infertile obese Pakistani women with PCOS (Rotterdam 2003 consensus)	119 (cases) were taking metformin 500 mg three times a day and continued throughout pregnancy. 70 conceived while on metformin only. 49 needed additional medications. Age 29 ± 4.1, BMI 32 ± 4.6.	In 78 cases, metformin was stopped in first trimester or they conceived without metformin. 21 conceived without medication, 13 conceived on metformin, and 44 required induction of ovulation and metformin. Age 26 ± 5.3, BMI 33.2 ± 5.2.	EPL (fetal loss before 12 weeks of gestation), gestational diabetes (GDM), and pregnancy induced hypertension (PIH), live births, intrauterine growth restriction (IUGR), and fetal anomalies.

28	59 nondiabetic infertile PCOS (Rotterdam criteria) patients with clomiphene citrate (CC) resistance and insulin resistance were conceived while taking metformin and different ovulation-inducing agents.	29 continued metformin throughout pregnancy. 1500 mg daily for BMI ≤29, 2000 mg daily for BMI 30–32 and 2500 mg daily for BMI >32. Age 28.14 ± 2.92, BMI 28.21 ± 2.37.	30 did not continue metformin throughout pregnancy. Age 26.13 ± 3.62, BMI 27.97 ± 2.49.	Abortion rate, development of GDM, live birth rate, congenital anomaly, macrosomia, and condition of newborn at birth.

29	137 infertile Pakistani women with PCOS (2003 Rotterdam Consensus criteria).	105 conceived while taking metformin. Group A (4–16 weeks met); group B (to 32 weeks met); group C (to delivery). Group A Group B Group C Age 28 ± 3.6 29 ± 3.1 27 ± 4.2 BMI 29.6 ± 5.1 30 ± 2.6 29.3 ± 3.3.	32 conceived without metformin Age 30 ± 2.9, BMI 31.2 ± 4.6.	PIH/preeclampsia; GDM; IUGR; miscarriage; preterm delivery; live birth; mean birth weight.

30	40 pregnant women (Norway) with PCOS (revised 2003 consensus) and without known diabetes mellitus were included in the first trimester.	22 took metformin 850 mg twice daily. Age 28.3 ± 1.7, BMI 29.3 ± 3.6.	18 with placebo. Age 28.9 ± 2.5, BMI 32.1 ± 3.1.	Fasting glucose, insulin levels, insulin resistance (HOMA-IR) and beta-cell function (HOMA-b) evaluated using the homeostasis assessment model and 2 h glucose levels during a standard 75 g OGTT, DM.

31	Nondiabetic women with PCOS (2003 European Society of Human Reproduction and Embryology—American Society for Reproductive Medicine diagnostic criteria)	Given 26% protein, 44% carbohydrate diets, without calorie restriction during pregnancy, metformin 2–2.55 g of metformin per day was taken in 120 pregnancies: 1700 mg/d in 6, 1500 mg in 39, 1000 mg/d in 6, and 750 mg/d in 1. Age 30 ± 5, BMI 33.5 ± 7.9.	47 women had at least one previous LB pregnancy (*n* = 64) without metformin.	The primary outcome measure was development of GD.

32	40 pregnant women with PCOS (Rotterdam ESHRE/ASRM sponsored PCOS workshop group, 2004).	18 women were randomized to metformin medication. Age 28.9 ± 4.8, BMI 32.1 ± 6.1.	22 were placebo. Age 28.3 ± 3.7, BMI 39.3 ± 8.0.	Androgen levels, pregnancy complications (preterm deliveries, preeclampsia/hypertension GD, CS, and ARDS). Infants (head circumference, birth-weight, birth-length, Apgar (5 min), and Apgar (10 min)).

33	72 women from the midwestern USA who were referred to a 1-year study of efficacy and safety of metformin therapy in PCOS.	Conceived on metformin, 1.5–2.55 g/day. BMI 33 ± 5.8.	Self as control	Gestational diabetes, number of first trimester SAB, live births, normal ongoing pregnancies ≥13 weeks, nature of intrauterine fetal development by sonography, congenital defects, infant birth weight and height, and height, weight, and motor and social development during the first 6 months of life.

34	1990 National Institutes of Health criteria	33 (32 white women and 1 Latina) nondiabetic women with PCOS who conceived while taking metformin and had live births; of these, 28 were taking metformin through delivery. Metformin, 2.55 g/d, throughout pregnancy in women with PCOS.	39 nondiabetic women with PCOS who had live birth pregnancies without metformin therapy.	Pretreatment height, weight, body mass index (BMI), glucose, insulin, insulin resistance and insulin secretion, and gestational diabetes.

35	40 pregnant women with PCOS (revised 2003 consensus' diagnostic criteria of PCOS) were recruited from the outpatient clinic at the University Hospital of Trondheim. All participants used two capsules once daily during the first week and two capsules twice daily for the rest of the pregnancy.	Treatment with metformin 425 mg (at 6–12, mean 8, gestational weeks). Age 28.9 ± 3.7, BMI 32.1 ± 6.1.	Identical placebo capsules. Age 28.3 ± 3.7, BMI 29.3 ± 8.0.	Minor complications included mild preeclampsia, hypertension and/or insulin-treated GDM. Severe complications included preterm deliveries before 32 gestational weeks, severe preeclampsia, or serious postpartum problems (e.g., endometritis and Group A streptococcal sepsis, adult acute respiratory distress syndrome (ARDS), thrombosis, or lung embolism).

36	57 infertile cases with PCOS (revised 2003 consensus' diagnostic criteria of PCOS) who became pregnant and were in the infertility unit and outpatient clinics in Zagazig university hospitals	Received metformin, starting in a dose of 1000 mg daily increased to 2500 mg daily according to BMI and response to treatment, some cases used other ovulation inducing drugs as clomiphene citrate and or gonadotrophines. When pregnancy occurred, cases continued on metformin in a dose of 1000–1500 mg daily till the end of pregnancy Age 30.2 ± 3.87, BMI 29.22 ± 2.31	Got pregnant spontaneously or by use of ovulation inducing agents but did not use metformin before or after pregnancy. Age 28.12 ± 4.35, BMI 28.35 ± 1.97	The rate of; spontaneous miscarriage, preterm delivery, fetal macrosomia, intrauterine growth restriction (IUGR), suspected fetal asphyxia at birth (5 min Apgar score 67) and recording of congenital malformation and neonatal mortality.

**Table 3:** Quality of the randomized controlled trials.

Number	Country	Allocation concealment	Blinding	ITT
		A: adequate	A: investigators	
		B: unclear	B: patients	
		C: inadequate	C: outcome assessors	
22	Norway (2 RCTs)	A	A: Yes B: Yes C: No	Yes

26	Norway (multicenter study)	A	A: Yes B: Yes C: Yes	No

30	Norway	A	A: Yes B: Yes C: Not reported	No

32	Norway	A	A: Yes B: Yes C: Not reported	No

35	Norway	A	A: Yes B: Yes C: Not reported	No

ITT: intention-to-treat.

**Table 4:** Findings of the trials included in the meta-analysis.

Number	Outcome	PCOS with metformin	Controls	Metformin versus controls
22	Second-trimester miscarriage and delivery <gestational week 37 + 0	5/153	18/159	*P* = 0.008
	Preeclampsia	12/153	7/157	*P* = 0.24
	New gestational diabetes	27/142	26/141	*P* = 0.90
25	GDM	8/200	32/160	*P* < 0.001, OR = 0.17, 95% CI = 0.07–0.37
	Gestational hypertension and/or preeclampsia	6/200	13/160	*P* = 0.03, OR = 0.35, 95% CI = 0.13–0.94
	Caesarean section rate	106/200	96/160	*P* = 0.18, OR = 0.75, 95% CI = 0.49–1.15
26	Preeclampsia	10/135	5/135	*P* = 0.18, RD = 3.7%, 95% CI = −1.7–9.2
	Preterm delivery	5/135	11/135	*P* = 0.12, RD = −4.4%, 95% CI = −10.1–1.2
	GDM	22/125	21/124	*P* = 0.87, RD = 0.8%, 95% CI = −8.6–10.2
				RD (risk difference)
	Weight	≤2500 g 8/135	≤2500 g 8/135	
		2501–4500 g 125/135	2501–4500 g 120/135	
		>4500 g 2/135	>4500 g 7/135	*P* = 0.32
	Length of delivery	50.3 ± 4.4	50.0 ± 2.5	*P* = 0.44
27	Miscarriage rate	9/119	23/78	*P* < 0.002
	EPL with recurrent miscarriage	2/16	5/11	*P* < 0.001
	Gestational diabetes	12/119	34/78	0.0021
	PIH	20/119	35/78	<0.002
	IUGR	20/119	30/78	<0.001
	Live birth rate	109/119	55/78	<0.001
28	GDM	1/29	9/30	OR = 12, 95% CI = 6.20–18.08
	Abortion	1/29	1/30	
	Macrosomia	0/29	4/30	
	LBW (low birth weight)	0/29	0/30	
	Birth asphyxia	0/29	5/30	
	Neonatal death	0/29	1/30	
	Preterm labor	2/29	3/30	
	Birth weight	2.79 ± 0.143	3 ± 0.499	*P* = 0.016
	APGAR (5 min)	10 ± 00	9.03 ± 0.326	*P* = 0.006
29		Group A Group B Group C		
	PIH/preeclampsia	14/32 6/18 6/45	8/26	*P* = 0.002
	GDM	15/32 10/18 13/45	12/26	*P* = 0.968
	IUGR	7/32 3/18 1/45	5/26	*P* = 0.026
	Miscarriage	8/40 2/20 0/45	6/32	*P* = 0.006
	Preterm delivery	8/32 5/18 2/41	5/26	*P* = 0.035
	Live birth	32/40 18/20 45/45	26/32	*P* = 0.016
	Mean birth weight	2.67 ± 0.912.71 ± 0.892.88 ± 0.95	2.9 ± 1.1	*P* = 0.81
30	GDM	6/22	3/18	
31	GDM	6/50	19/64	
32	Preterm deliveries	0/18	5/22	
	Preeclampsia/hypertension	3/18	2/22	
	GDM	1/18	3/22	
	CS (caesarean section)	3/18	4/22	
	Head circumference	36 ± 1	34 ± 5	*P* = 0.07
	Birth weight	3595 ± 420	3215 ± 1048	*P* = 0.1
	Birth length	50 ± 2	48 ± 8	*P* = 0.2
	Apgar (5 min)	9.3 ± 1.0	9.5 ± 0.6	*P* = 0.3
	Apgar (10 min)	9.8 ± 0.7	9.9 ± 0.2	*P* = 0.3
33	SAB (first-trimester spontaneous abortion)	12/46	62/100	McNemar's = 32, df = 1, *P* < 0.0001
	GDM	3/68	9/34	McNemar's = 5, *P* = 0.025
34	GDM	1/33	10/37	*P* = 0.0074 (Fisher test)
35	GDM	2/18	6/22	*P* = 0.3
	Overall pregnancy complications	3/18	10/22	*P* = 0.09
	Severe pregnancy complications	0/18	7/22	*P* = 0.01
36	Miscarriage	1/31	7/26	*P* = 0.01
	Preterm birth	1/31	2/26	*P* = 0.58
	Fatal macrosomia	1/31	1/26	*P* = 1.0
	IUGR	0/31	1/26	*P* = 0.45
	5 min Apgar score (≤7)	1/31	3/26	*P* = 0.32
	Fatal anomalies	0/31	1/26	*P* = 0.45
	Neonatal mortality	0/31	1/26	*P* = 0.45
	GDM	1/31	6/26	
